# BCS Class IV Oral Drugs and Absorption Windows: Regional-Dependent Intestinal Permeability of Furosemide

**DOI:** 10.3390/pharmaceutics12121175

**Published:** 2020-12-02

**Authors:** Milica Markovic, Moran Zur, Inna Ragatsky, Sandra Cvijić, Arik Dahan

**Affiliations:** 1 Department of Clinical Pharmacology, School of Pharmacy, Faculty of Health Sciences, Ben-Gurion University of the Negev, Beer-Sheva 8410501, Israel; milica@post.bgu.ac.il (M.M.); moranfa@post.bgu.ac.il (M.Z.); inna.ragatsky@gmail.com (I.R.); 2 Department of Pharmaceutical Technology and Cosmetology, Faculty of Pharmacy, University of Belgrade, Vojvode Stepe 450, 11221 Belgrade, Serbia; sandra.cvijic@pharmacy.bg.ac.rs

**Keywords:** BCS class IV drugs, segmental-dependent intestinal permeability, intestinal absorption, oral drug delivery, biopharmaceutics, physiologically-based pharmacokinetic (PBPK) modeling, furosemide

## Abstract

Biopharmaceutical classification system (BCS) class IV drugs (low-solubility low-permeability) are generally poor drug candidates, yet, ~5% of oral drugs on the market belong to this class. While solubility is often predictable, intestinal permeability is rather complicated and highly dependent on many biochemical/physiological parameters. In this work, we investigated the solubility/permeability of BCS class IV drug, furosemide, considering the complexity of the entire small intestine (SI). Furosemide solubility, physicochemical properties, and intestinal permeability were thoroughly investigated in-vitro and in-vivo throughout the SI. In addition, advanced in-silico simulations (GastroPlus^®^) were used to elucidate furosemide regional-dependent absorption pattern. Metoprolol was used as the low/high permeability class boundary. Furosemide was found to be a low-solubility compound. Log D of furosemide at the three pH values 6.5, 7.0, and 7.5 (representing the conditions throughout the SI) showed a downward trend. Similarly, segmental-dependent in-vivo intestinal permeability was revealed; as the intestinal region becomes progressively distal, and the pH gradually increases, the permeability of furosemide significantly decreased. The opposite trend was evident for metoprolol. Theoretical physicochemical analysis based on ionization, pK_a_, and partitioning predicted the same trend and confirmed the experimental results. Computational simulations clearly showed the effect of furosemide’s regional-dependent permeability on its absorption, as well as the critical role of the drug’s absorption window on the overall bioavailability. The data reveals the absorption window of furosemide in the proximal SI, allowing adequate absorption and consequent effect, despite its class IV characteristics. Nevertheless, this absorption window so early on in the SI rules out the suitability of controlled-release furosemide formulations, as confirmed by the in-silico results. The potential link between segmental-dependent intestinal permeability and adequate oral absorption of BCS Class IV drugs may aid to develop challenging drugs as successful oral products.

## 1. Introduction

The biopharmaceutical classification system (BCS) developed by Amidon et al. revealed that the solubility/dissolution of the drug and its intestinal permeability are the two key factors that dictate drug absorption following oral administration [1,2]. Drug solubility in the gastrointestinal milieu may change in different intestinal segments, e.g., due to pH changes, in a fairly predictable manner; depending on the pKa, the solubility of acidic drugs may increase as the luminal pH rises in more distal regions of the small intestine, and vice versa for basic drugs [3,4,5]. On the other hand, time- and segmental-dependent intestinal permeability is more complicated and harder to predict [1]. Mechanisms contributing to segmental-dependent permeability throughout the gastrointestinal tract (GIT) include different morphology along the GIT, variable intestinal mucosal cell differentiation, changes in the drug concentration (in case of carrier-mediated transport), modulation of tight junction permeability, and luminal contents and properties, e.g., pH, transporter expression, variability in the structure/composition of the intestinal membrane itself, and more [6,7,8,9,10,11].

The four BCS classes highlight the limiting factors of the absorption process: (1) Class I, high-solubility high-permeability drugs, indicate the easier and straightforward development process, and complete absorption is expected; (2) Class II, low-solubility high-permeability drugs, indicate that a solubility/dissolution limitation is expected; (3) Class III, high-solubility low-permeability drugs, indicate that the intestinal absorption of this class of drugs will be limited by the permeability rate; and (4) Class IV, low-solubility low-permeability drugs [12]. Since Class IV drugs suffer from inadequate solubility and permeability, they have very poor oral bioavailability and are inclined to exhibit very large inter- and intrasubject variability. Therefore, unless the drug dose is very low, they are generally poor oral drug candidates. Yet, according to some estimates, ~5% of the world’s top oral drugs belong to this class [13,14,15]. In some cases, this is due to the absorption window, which is often critical for the success or failure of a certain drug. In order to gather information about the drug absorption window, extensive work and thorough analysis of luminal conditions and drug absorption is needed, within different locations throughout the GIT. Here, we present such analysis for BCS class IV drug, furosemide [16].

Furosemide is a powerful loop diuretic and is indicated for treating edematous conditions associated with heart, renal, and hepatic failure, as well as for the treatment of hypertension [17,18]. Drug therapy with furosemide is often complex, due to apparent erratic oral systemic availability and unpredictable responses to an administered dose [19]. Even though furosemide is a class IV drug, it is a very common and widely prescribed drug on the market.

In this work, we aimed to investigate the reason for apparent success of furosemide as a marketed product, despite its poor biopharmaceutical properties, and classification as BCS class IV drug, in order to allow development of future class IV compounds. We posit that segmental-dependent permeability of furosemide may contribute to its absorption complexity and provide a certain absorption window in which the drug has suitable permeability and, hence, gets absorbed. For this reason, we investigated the in-vivo intestinal permeability of furosemide throughout different segments of the small intestine. Solubility studies, as well as theoretical physicochemical analysis of furosemide and advanced modern in-silico GastroPlus^®^ simulations, were performed, in order to elucidate the mechanistic reasons behind the experimental results. Furosemide data were compared to the β-blocker metoprolol, the Food and Drug Administration (FDA) reference drug for the low/high permeability class boundary. Overall, this experimental setup allowed us to reveal important insights on the performance of furosemide, despite its unfavorable drug-like properties, and discuss extrapolation of these insights to other BCS class IV drug candidates.

## 2. Methods

### 2.1. Materials

Furosemide, metoprolol, phenol red, potassium chloride, potassium phosphate monobasic, potassium phosphate dibasic, sodium chloride, acetic acid, maleic acid, *n*-octanol, and trifluoroacetic acid (TFA) were all purchased from Sigma Chemical Co. (St. Louis, MO, USA). Acetonitrile and water, ultra-performance liquid chromatography (UPLC) grade were purchased from Merck KGaA, Darmstadt, Germany. Remaining chemicals were of analytical reagent grade.

### 2.2. Solubility Studies

The pH-dependent solubility studies were performed using the shake flask method, as previously reported [20,21,22,23]. The equilibrium solubility of furosemide was determined at both 37 °C and at room temperature (25 °C), in phosphate buffer pH 7.5, acetate buffer pH 4.0, and maleate buffer pH 1.0. Surplus quantity of furosemide was introduced to glass vials holding buffer solutions with different pH; the pH of those solutions was measured following drug addition to the buffers and, consequently, placed in the shaking incubator (100 rpm) at 37 °C. The vials were centrifuged (10,000 rpm, 10 min), and the supernatant was instantly analyzed by UPLC. The dose number for furosemide was calculated using the established equation: *D*_0_ = *M*/*V*_0_/*C*_s_; *M* being the highest single-unit dose strength of furosemide (taken as 80 mg [24]), *V*_0_ is the initial volume of water (250 mL), and *C*_s_ is the solubility at each pH; the drug is considered highly soluble if the *D*_0_ < 1.

### 2.3. Evaluation of Octanol-Buffer Partition Coefficients (Log D)

Furosemide and metoprolol experimental octanol-buffer partition coefficients (Log D) were studied at pH 6.5, 7.0, and 7.5 using the shake-flask method [8,11]. Drug solutions in octanol-saturated phosphate buffer (pH 6.5, 7.0, 7.5) were equilibrated at 37 °C for 48 h. The octanol and water phase were divided via centrifugation, and the drug content in the water phase was quantified using UPLC; the furosemide/metoprolol concentration in the octanol phase was determined by mass balance.

### 2.4. Physicochemical Analysis

The theoretical fraction extracted into octanol (f_e_) was calculated using the following equation [25,26].

$$f_{e}=\frac{f_{u}P}{1\;+{\;f}_{u}P},$$

in which P represents the octanol-water partition coefficient of the unionized drug form, and f_u_ is the fraction unionized of the drug at a certain pH. Experimental Log P values were taken from the literature for both furosemide (2.29) [27] and metoprolol (2.19) [28]. The f_u_ versus pH was plotted according to the Henderson-Hasselbalch equation, using the pK_a_ literature values: 9.68 for metoprolol [29] and 3.8 for furosemide [24].

### 2.5. Rat Single-Pass Intestinal Perfusion

Effective permeability coefficient (P_eff_) of furosemide versus metoprolol in various intestinal segments was assessed using the single-pass rat intestinal perfusion (SPIP) in-vivo model. The murine studies were completed according to the approved protocol by Ben-Gurion University of the Negev Animal Use and Care Committee (Protocol IL-08-01-2015). The animals (male Wistar rats weighing 230–260 g, Harlan, Israel) were housed and handled according to Ben-Gurion University of the Negev Unit for Laboratory Animal Medicine Guidelines. All animals were fasted overnight (12–18 h) with free access to water; rats were randomly allocated to different experimental groups. The intestinal perfusion study was performed according to the previous reports [7,9,30,31,32]. Animals were anesthetized via intramuscular injection of 1 mL/kg ketamine-xylazine solution (9%:1%) and placed on a heated (37 °C) surface (Harvard Apparatus Inc., Holliston, MA, USA); the rat abdomen was uncovered via a midline incision (~3 cm). Permeability (P_eff_) was measured in proximal jejunum (starting 2 cm lower from the ligament of Treitz), mid-small intestine (SI) segment (isolated between the end of the upper and the beginning of the lower segments), and distal segment of the ileum (ending 2 cm above the cecum) accounting for the complexity of the entire SI [7]. Intestinal segments were cannulated on both ends and perfused with drug-free buffer. Working solutions containing furosemide (320 µg/mL), metoprolol (400 µg/mL), and phenol red (a non-absorbable marker for water flux measurements) were prepared with potassium phosphate monobasic and sodium phosphate dibasic, to achieve pH of 6.5, 7.0 and 7.5; osmolarity (290 mOsm/L) and ionic strength in all buffers was maintained throughout the study. Drug solutions were incubated in a 37 °C water bath. Steady-state environment was ensured by perfusing the drug-containing buffer for 1 h, followed by additional 1 h of perfusion, during which sampling was done every 10 min. The pH of the collected samples was measured in the outlet sample to verify that there was no pH change throughout the perfusion. All samples were assayed by UPLC. The length of each perfused intestinal segment was measured in the end of the experiment. The effective permeability (P_eff_; cm/s) through the rat SI wall was calculated according to the following equation:

$$P_{\mathrm{eff}}=\frac{-\mathrm{Qln}\;({C^{\prime }}_{\mathrm{out}}/{C^{\prime }}_{\mathrm{in}})}{2\pi \mathrm{RL}},$$

in which Q is the perfusion buffer flow rate (0.2 mL/min); ${C^{\prime }}_{\mathrm{out}}/{C^{\prime }}_{\mathrm{in}}$ is the ratio of the outlet/inlet drug concentration adjusted for water transport; R is the radius of the intestinal segment (conventionally used as 0.2 cm); and L is the exact length of the perfused SI segment as was measured at the experiment endpoint [7,33,34].

### 2.6. Analytical Methods

Concentration of furosemide and metoprolol was evaluated using an UPLC instrument Waters Acquity UPLC H-Class (Milford, MA, USA), with a photodiode array detector and Empower software. Furosemide and metoprolol were separated on Acquity UPLC XTerra C18 3.5 µm 4.6 mm × 250 mm column (Waters Co., Milford, MA, USA). Gradient mobile phase, going from 70:30% to 90:10% *v/v* 0.1% trifluoroacetic acid in water/acetonitrile, respectively, on a flow rate of 1 mL/min (25 °C). The inter- and intraday coefficients of variation were < 1.0% and 0.5%, respectively.

### 2.7. Statistics

Solubility studies were performed in four replicates; Log D studies were performed in six replicates, whereas animal perfusion studies were *n* = 4. Values are expressed as means ± standard deviation (SD). To determine statistically significant differences among the experimental groups, a 2-tailed nonparametric Mann–Whitney U test for 2-group comparison was used; *p* < 0.05 was termed significant.

### 2.8. In-Silico Simulations

Computer simulations of furosemide absorption and concomitant plasma concentrations following oral administration in humans were conducted using GastroPlus^TM^ software package (v. 9.7.0009, 2019, Simulations Plus Inc., Lancaster, CA, USA). The required input data regarding drug physicochemical and pharmacokinetic properties were experimentally determined, taken from literature or in-silico predicted. Human permeability values throughout the SI were calculated from the experimental rat single-pass intestinal perfusion data, using the software integrated “permeability converter”. Drug disposition was best described by three-compartmental pharmacokinetic model, whereas the relevant parameters (clearance (*CL*), volume of distribution (*V*d) and distribution constants between central and peripheral compartments) were estimated using PKPlus software module, based on the in-vivo plasma concentration data for an intravenous (i.v.) bolus dose [35]. The application of three-compartmental model to describe furosemide pharmacokinetics has already been reported in literature [36,37]. Graphical data from literature were digitized using DigIt™ program (version 1.0.4, 2001–2008, Simulations Plus, Inc., Lancaster, CA, USA). Physiological parameters were the software default values representing fasted state physiology of a healthy human representative.

The software simulates drug absorption from the GIT using the integrated Advanced Compartment Absorption and Transit (ACAT) GIT model that consists of nine compartments (stomach, duodenum, two segments of jejunum, three segments of ileum, caecum, and ascendant colon). These compartments are linked in series, and the amount of drug dissolved and absorbed from each compartment is calculated by the system of differential equations. More details on the ACAT model can be found in the literature [38,39]. Regarding the fact that furosemide is a poorly-soluble drug, the model accounted for the effect of bile salt on drug solubility and diffusion coefficient. Drug dissolution rate under physiological conditions was predicted using the software default Johnson dissolution equation (based on modified Nernst-Bruner equation) [40].

The validity of the model (i.e., the selection of input values) was validated by comparison of the prediction results (bioavailability (F), maximum plasma concentration (C_max_), time to reach C_max_ (t_max_), and area under the plasma concentration-time curve (AUC_0–∞_)) with published data from the in-vivo studies for peroral (p.o.) drug administration. Percent prediction error (%PE) between the predicted and mean in-vivo observed data from a clinical study was calculated using the following equation:

$$\%\mathrm{PE}=\frac{\left(\mathrm{Observed}\;\mathrm{value}-\mathrm{Predicted}\;\mathrm{value}\right)\times 100}{\mathrm{Observed}\;\mathrm{value}}.$$

In the next step, the generated model was used to mechanistically interpret furosemide regional absorption pattern, and to estimate the outcomes for various hypothetical drug dissolution scenarios (illustrating drug dissolution from immediate-release (IR) and controlled-release (CR) oral formulations). In the last case, hypothetical dissolution profiles were used as additional inputs to describe drug release rate in-vivo, and the selected dosage form was “CR dispersed” to allow input of the tabulated dissolution data.

## 3. Results

The solubility values obtained for furosemide at 37 °C and at room temperature (25 °C) are summarized in Table 1, as well as the corresponding dose number (D_0_). Furosemide showed pH-dependent solubility, in accordance with its acidic nature. It can be seen that, while, at pH 7.5, furosemide has suitable solubility (as evident by D_0_ lower than 1), at the lower pH values, 1.0 and 4.0, it is poorly soluble. When taking 80 mg as the highest dose strength, although D_0_ < 1 was obtained at pH 7.5, at pH 1.0 and 4.0, the D_0_ is higher than 1; hence, furosemide was found to be a low-solubility compound according to the BCS.

**Figure 1:**
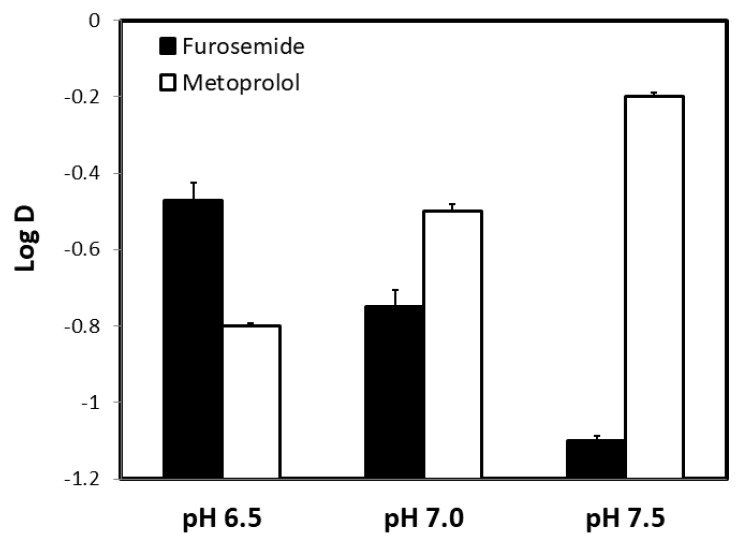
The octanol-buffer partition coefficients, Log D, for furosemide and metoprolol at the three pH values 6.5, 7.0, and 7.5. Data are presented as the mean ± S.D.; *n* = 6 in each experimental group.

Octanol-buffer partition coefficient values of furosemide and metoprolol at the three pH values 6.5, 7.0, and 7.5 (representing the conditions throughout the small intestine) are presented in Figure 1. Both drugs presented a clear pH-dependent Log D values across the studied pH range, with opposite trends; while furosemide’s partitioning decreases as the pH rises, metoprolol shows higher partitioning into octanol at higher pH (metoprolol is the acceptable reference drug for the low/high permeability class boundary). In addition, furosemide’s Log D at pH 6.5 was higher than that of metoprolol at the same pH; this is a surprising finding since Log D may sometimes be used as a surrogate for passive permeability. Indeed, at higher pH values (7.0 and 7.5), metoprolol Log D increases, while furosemide decreases, and metoprolol Log D becomes higher than furosemide.

**Figure 2:**
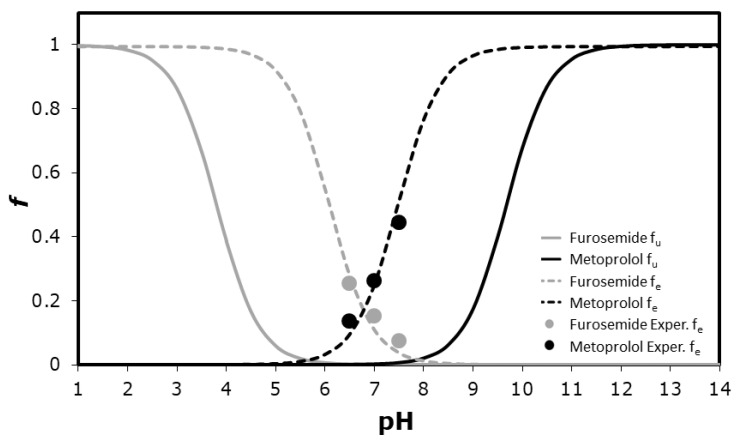
The theoretical fraction unionized (f_u_) and fraction extracted into octanol (f_e_) plots as a function of pH for furosemide and metoprolol, as well as experimental buffer-octanol partitioning of the drugs in the three pH values 6.5, 7.0, and 7.5 (*n* = 5).

Furosemide and metoprolol physicochemical properties are presented in Table 2. Figure 2 presents furosemide versus metoprolol theoretical fraction unionized (f_u_) and fraction extracted into octanol (f_e_) as a function of pH. The plots have a standard sigmoidal shape, with opposite trends for furosemide vs. metoprolol. The f_e_ vs. pH plot follows the same pattern to the f_u_ plot, only with a shift to the right (higher pH values) for acidic drug (furosemide), and to the left (lower pH values) for basic (metoprolol) drugs. The shift magnitude in both cases equals Log(P − 1) at the midpoint of the f_e_ and f_u_ curves [25,26]. The experimental drug octanol-buffer partitioning at the three pH values (6.5, 7.0, and 7.5) are illustrated in Figure 2, as well, and it can be seen that they were in excellent agreement with the theoretical plots.

**Figure 3:**
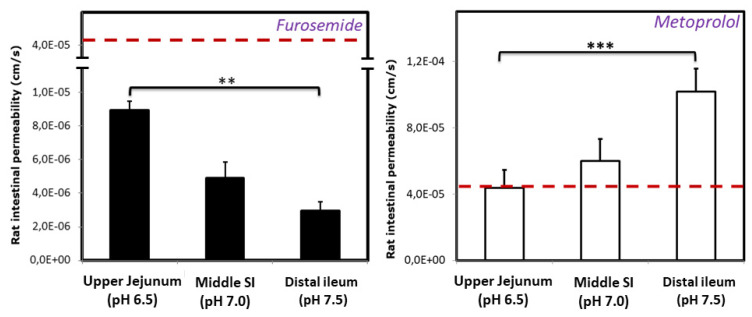
Effective permeability values (P_eff_; cm/s) obtained for furosemide and metoprolol after in-situ single pass perfusion to the rat proximal jejunum at pH 6.5, mid-small intestine at pH 7.0, and to the distal ileum at pH 7.5. Mean ± S.D.; *n* = 4 in each experimental group; ** *p* < 0.01, *** *p* < 0.001.

The effective permeability coefficient (P_eff_, cm/sec) values of furosemide and metoprolol determined using the single-pass intestinal perfusion (SPIP) rat model, in three intestinal segments, namely proximal jejunum (pH 6.5), mid small intestine (pH 7.0), and distal ileum (pH 7.5), are presented in Figure 3. It can be seen that significant regional-dependent permeability of furosemide throughout the small intestine was evident: the permeability of furosemide gradually decreases, while the permeability of metoprolol gradually increases, as the SI segments become more distal.

**Figure 4:**
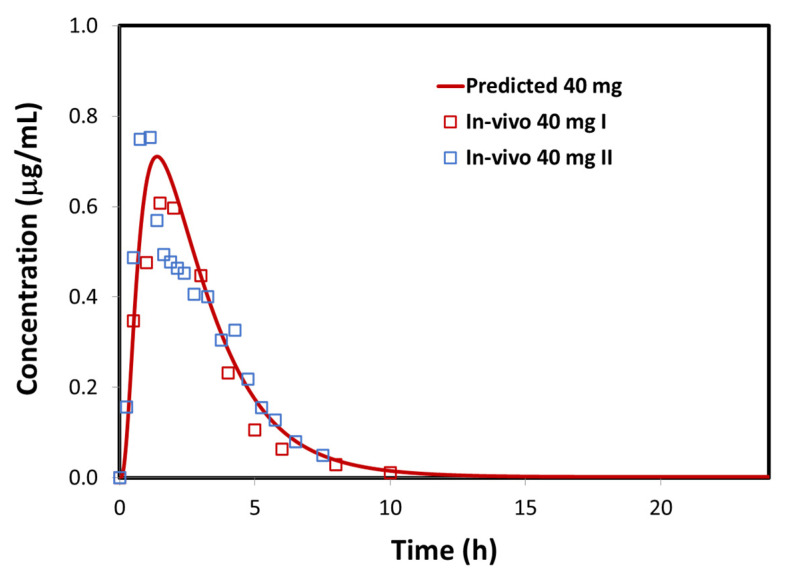
GastroPlus^®^ simulated (line) versus mean observed (markers) plasma concentration profiles following p.o. administration of furosemide. Mean observed values represent 40 mg immediate-release (IR) tablet profile I [43] and 40 mg IR tablet profile II [37].

The input data regarding drug physicochemical and pharmacokinetic properties, used for in-silico simulations, are presented in Table 3. The simulated furosemide plasma concentration profile following p.o. administration is depicted in Figure 4, along with the mean profiles observed in the in-vivo studies. In addition, the observed and model predicted pharmacokinetic parameters are compared in Table 4. The presented data demonstrate that the generated model adequately describes furosemide absorption and disposition. The course of the predicted plasma profile fairly resembles the observed data. However, certain variations are observed between the mean in-vivo data from different studies referring to the same drug dose (Figure 4, Table 4). Indeed, it has been reported that furosemide oral absorption is highly variable between individuals, e.g., C_max_ varied three-fold, and t_max_ varied five-fold [36,37,41]; moreover, individual AUC values for 40 mg furosemide oral dose varied between 1.57 and 3.76 µg∙h/mL (more than two-fold) [36,37,41], and even larger AUC values were observed in another study with the same drug dose (2.23–6.10 µg∙h/mL) [42], indicating that, regardless of the high PE(%) values in Table 4, the model predicted value of 3.66 µg∙h/mL is not an overestimate of the extent of drug absorption. In addition, extensive intrasubject variability was observed for orally dosed furosemide, and these variations were attributed to the absorption process (i.e., day to day variations in physiological factors) since the repeated i.v. doses showed only marginal intrasubject variability [36,37,41]. Considering pronounced inter- and intraindividual variability in furosemide oral absorption, the simulated profile can be seen as a reasonable estimate (Figure 4). Moreover, the predicted fraction of oral drug absorption (cc. 52%) is in accordance with the values reported in the literature [36,37].

**Figure 5:**
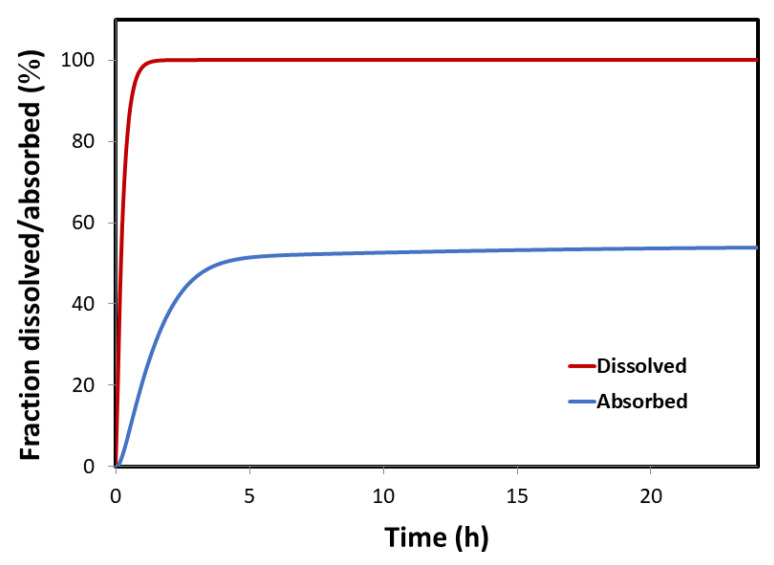
GastroPlus^®^ simulated dissolution and absorption profiles following p.o. administration of 40 mg furosemide dose (dissolution profile was simulated using the software default Johnson equation).

**Figure 6:**
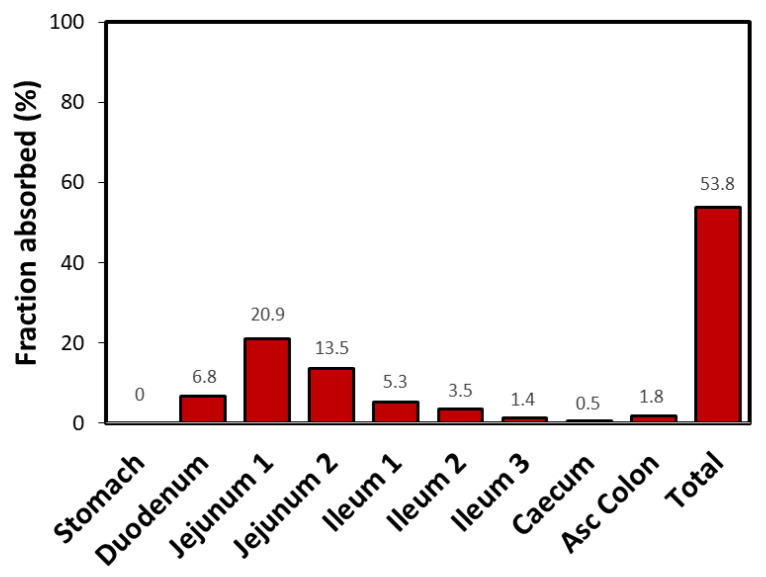
GastroPlus^®^ simulated regional absorption of furosemide following p.o. administration of 40 mg drug dose (the simulated values refer to the fraction of drug dose that entered into the enterocytes).

The predicted furosemide dissolution and absorption profiles following an IR oral formulation (IR tablet) are illustrated in Figure 5. The generated profiles clearly indicate that drug permeability is the limiting factor for absorption under fasted state GIT conditions. Namely, although furosemide is a low-solubility drug, due to ionization at the elevated pH conditions in the proximal SI, drug dissolution from an IR formulation is expected to be fast (>85% in 30 min). Therefore, furosemide absorption from an IR formulation is mainly governed by poor permeability. The predicted regional-dependent absorption distribution (Figure 6) further highlights the role of furosemide segmental absorption on the overall drug bioavailability. As implied by the regional-dependent permeability data, but also considering the surface area available for absorption, furosemide absorption predominantly happens in the proximal parts of the SI (76.6% of the total amount absorbed into the enterocytes), and only a minor fraction of drug (23.2% of the total amount absorbed into the enterocytes) passes into systemic circulation through mid and distal GIT regions.

**Figure 7:**
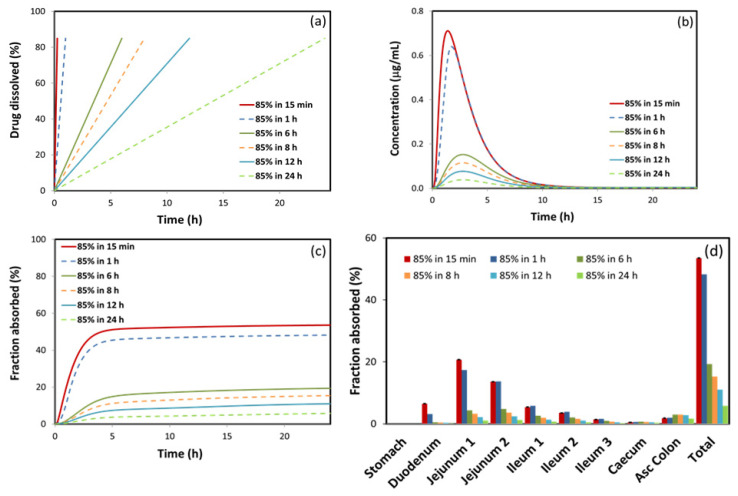
GastroPlus^®^ simulated furosemide dissolution profiles (**a**); and (**b**) the corresponding simulated plasma profiles; (**c**) absorption profiles; and (**d**) regional absorption distribution.

The prediction results corresponding to various dissolution scenarios are presented in Figure 7b–d and Table 5. According to the simulated data, furosemide release rate from an oral formulation highly impacts the concomitant absorption process, whereas prolonged drug release rate leads to marked delay in the rate and extent of drug absorption. The estimated pharmacokinetic parameters (Table 5) indicate that furosemide bioavailability would show more than a 10-fold decrease in case the complete drug dissolution is achieved within 24 h in comparison to 15 min. A similar trend is observed for C_max_ and AUC values (17.75- and 17.38-fold decrease, respectively), while t_max_ would be prolonged (about two-fold). It is interesting to note that t_max_ increases with decrease in drug dissolution up to some point, but further decrease in drug dissolution (e.g., 85% in more than 6 h) would not cause additional delay in peak plasma concentration. This is because, after cc. 2 h, the drug leaves proximal parts of the intestine, where majority of furosemide absorption takes place, and, later on, in mid and especially distal intestine, only a small fraction of drug can be absorbed, as illustrated in Figure 7d.

## 4. Discussion

BCS class IV drugs (e.g., sulfamethoxazole, ritonavir, paclitaxel, and furosemide) exhibit numerous unfavorable characteristics (low solubility and permeability, high presystemic metabolism, efflux transport), which make their oral drug delivery challenging. In addition to this, class IV drugs often demonstrate inter/intra-subject variability. Indeed, following oral administration, the absorption and bioavailability of furosemide are highly variable (37–51%) [35,41]. It has been suggested that this variability is highly depend on the absorption process [41], which in turn is dependent on drug aqueous solubility and intestinal permeability following oral administration [1,44]. It has also been hypothesized that variable gastric/intestinal first-pass metabolism can be a factor in causing incomplete and irregular furosemide absorption in humans [45]. Despite the unfavorable class IV drug characteristics, furosemide was shown to be exceptionally useful and successful marketed drug product for the treatment of edema [17]. For this reason, we decided to investigate furosemide’s solubility and in-vivo regional-dependent permeability throughout the GIT, as main parameters that guide absorption of oral drugs.

It was shown that a correlation between human P_eff_ in the jejunum and physicochemical parameters advocates that there is a high pH-dependent influence on the passive intestinal permeability in-vivo [46]. Indeed, furosemide in-vivo permeability data demonstrate a downward trend towards the distal intestinal segments as the pH gradually increases, a trend that can be expected for acidic drugs, since the pH in the intestinal lumen gradually increases towards distal SI regions (Figure 3). Many BCS class IV drugs are substrates for efflux transporters [47]. There is some evidence that furosemide might be a substrate for efflux transporters [48,49]; thus, such permeability trend could also be influenced by the P-glycoprotein (P-gp) transporter in which expression levels are increased from proximal to distal SI segments [6,50,51,52]. Since metoprolol’s intestinal permeability is passive and does not involve carrier-mediated absorption, it exhibited pH-dependent intestinal permeability, with reverse tendency compared to furosemide; as a basic drug, metoprolol showed upward increase in permeability towards distal SI segments with rising pH values (Figure 3). At any point throughout the SI, furosemide exhibited significantly lower permeability than the benchmark (metoprolol’s jejunum permeability), which confirms its BCS low-permeability classification and incomplete absorption. Despite the fact that furosemide is a low-permeability drug, the higher permeability in the proximal intestinal regions provides a window for furosemide absorption, and we posit that this is one of the main reasons for furosemide’s sufficient bioavailability and success as a marketed drug. Theoretical f_u_ and f_e_ as a function of pH were found to be in excellent correlation to these in-vivo data. In addition, in-silico modeling indicated that furosemide dissolution from an IR formulation would be fairly complete before the drug leaves proximal SI (Figure 5), although the drug is generally classified as low-soluble, enabling timely delivery of the dissolved drug to the distinct absorption site. Complete furosemide dissolution under physiological conditions is also confirmed by the experimental solubility results (Table 1).

Furosemide Log D studies showed higher partition coefficient in comparison to metoprolol at pH 6.5, whereas, in the in-vivo intestinal perfusion experiment, furosemide showed significantly lower jejunum permeability than metoprolol (Figure 1). A possible reason for this difference in the partitioning and in-vivo permeability can be the polar surface area (PSA) of both drugs [53]. A sigmoidal relationship between the fraction absorbed following oral administration and the dynamic polar surface area was reported in the past [54,55,56]. It was shown that orally administered drugs with large PSA (>120) are hardly absorbed by the passive transcellular route, while drugs with a small PSA (<60) are almost completely absorbed [55,56]. This is in agreement with our results, as furosemide has much higher PSA (127.7) than metoprolol (53.2) [54,55]. Another reason for the difference in the partitioning and in-vivo permeability may be the presence of active efflux transport involved in the intestinal permeability. The influence of efflux transport at pH 6.5 (proximal intestinal segments) could decrease furosemide’s permeability in-vivo, which was not accounted for in the octanol partitioning studies.

The Log P value of furosemide (2.3) is in the close proximity to that of metoprolol (2.2), pointing to high permeability (Table 2). However, the Log P calculation is based on the unionized drug fraction, and, since furosemide has acidic nature it is likely that, once it passes the acidic stomach environment, it will mostly be in ionized form (the pH throughout the GIT varies from 5.9–6.3 in the proximal SI to 7.4–7.8 in distal SI segments; pH in the colon is fluctuating between pH 5–8 [57]); therefore the high furosemide Log P is not in correspondence with permeability in-vivo. Thus, we posit that no single parameter can be used for measuring the drug absorption process, but rather, a combination of physicochemical parameters and in-vitro and in-vivo findings, as well as careful consideration of inclusion criteria prior to making decisions. Despite the high Log P value for furosemide, it was indeed confirmed that furosemide is a BCS class IV drug, based on both the solubility data (Table 1) and the intestinal permeability (Figure 3).

Suitable formulation is the main approach to create an efficacious drug product for the administration of BCS class IV drugs [47]. Absorption windows in the proximal intestinal segments can restrict the oral drug bioavailability and can be a significant limitation for the development of CR drug formulation. The underlying reasons are mechanistically explained by our in-silico results (Figure 7). As mentioned, furosemide permeability results revealed acceptable permeability in the proximal segments of the SI, which is presumably the reason why furosemide has appropriate drug bioavailability, despite being a BCS class IV drug. However, since CR products release the drug over 12–24 h, mostly in the colon, (transit time throughout the small intestine is 3–4 h [58]), the fact that furosemide is mainly absorbed from proximal SI segments, (with decreased permeability at distant GIT segments) prevents the formulation of furosemide as a CR product, as shown previously [21,59,60]. However, we believe that formulations based on gastro-retentive dosage forms (GRDF) can be shown as prosperous for furosemide [61]. There are several similar examples in the literature where absorption window occurs in the upper GI, and this has been used to create GDRF formulations to improve the drug absorption, such as riboflavin [62] and levodopa [59,63].

Several types of bariatric surgeries (specifically Roux-en-Y gastric bypass and mini bypass) result in bypassing the upper SI. In cases where the absorption window is indeed in this upper SI region, the absorption following the bariatric surgery can be hampered vastly, since the actual segment responsible for the majority of absorption is bypassed [64,65,66].

## 5. Conclusions

Regional-dependent permeability throughout the small intestine was evident for furosemide. The permeability of furosemide gradually decreases throughout the small intestine as a function of the pH change in the intestinal lumen. However, at any point throughout the small intestine, furosemide exhibited significantly lower permeability than the benchmark of metoprolol′s permeability in the jejunum, which may explain the incomplete absorption of the drug. We propose that, for a drug to be classified as BCS low-permeability, its intestinal permeability should not match/exceed the low/high class benchmark anywhere throughout the intestinal tract, as well as is not restricted necessarily to the jejunum. Nevertheless, low-permeable drugs should not be treated as ‘unfavorable’ by default; instead, therapeutic potential and suitable formulation strategies should be considered on a case-by-case basis, taking into account the overall results of in-vitro, in-vivo, and in-silico testing, throughout the entire gastrointestinal tract.

## Figures and Tables

**Table 1:** Furosemide solubility values (µg/mL) at the tree pH values 1.0, 4.0, and 7.5, at 37 °C (upper panel), and at room temperature (25 °C; lower panel), as well as the corresponding dose number (D_0_) calculated for an 80-mg dose. Data presented as mean ± SD; *n* = 6.

**At 37 °C**		
**pH**	**Solubility (µg/mL)**	**Corresponding D_0_**
1	19.4 ± 3.7	16.5
4	65.5 ± 9.0	4.8
7.5	8340.1 ± 81.6	0.04
**At 25 °C**		
**pH**	**Solubility (µg/mL)**	**Corresponding D_0_**
1	40.3 ± 16.2	7.9
4	56.7 ± 12.2	5.6
7.5	8550.6 ± 149.4	0.04

**Table 2:** Physicochemical parameters and chemical structure of furosemide and metoprolol.

Drug	Chemical Structure	pK_a_	Log P	PSA
Furosemide	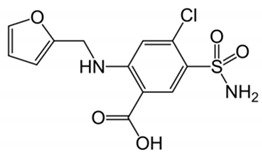	3.8	2.3	127.7
Metoprolol	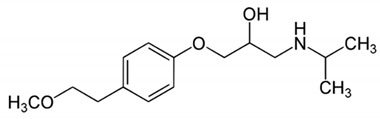	9.7	2.2	53.2

**Table 3:** The selected input parameters for furosemide absorption GastroPlus^®^ simulation.

Parameter	Value	Source
Molecular weight (g/mol)	330.75	/
Log D (pH 7.5)	−1.0818	experimental values
Solubility at 37 °C (µg/mL)	19.4 (pH 1.0)	
	65.5 (pH 4.0)	
	8340.1 (pH 7.5)	
pK_a_ (acid)	3.8	[24]
Human effective permeability, P_eff_ (cm/s)	0.4043 × 10^−4^ (duodenum, jejunum)	values converted using GastroPlus™ integrated “permeability converter” based on experimental rat perfusion data
	0.2246 × 10^−4^ (ileum 1 and 2)	
	0.1392 × 10^−4^ (ileum 3, caecum, colon)	
Diffusion coefficient (cm^2^/s)	0.7289 × 10^−5^	GastroPlus™ calculated value (based on molecular weight)
Mean precipitation time (s)	900	GastroPlus™ default values
Particle density (g/mL)	1.2	
Particle radius (µm)	25	
Blood/plasma concentration ratio	1	
Plasma fraction unbound (%)	1	[24]
Clearance, CL (L/h/kg)	0.121	calculated using GastroPlus™ PKPlus module, based on the i.v. data [35]
Volume of distribution, Vd (L/kg)	0.043	
Distribution constant k_12_ (1/h)	0.964	
Distribution constant k_21_ (1/h)	1.614	
Distribution constant k_13_ (1/h)	0.925	
Distribution constant k_32_ (1/h)	0.708	
Regional pH in the GIT	1.3; 6.0; 6.2; 6.4; 6.6; 6.9; 7.4; 6.4; 6.8	GastroPlus™ default values for stomach, duodenum, jejunum 1, jejunum 2, ileum 1, ileum 2, ileum 3, caecum, and ascendant colon
Regional volume of fluid in the GIT (mL)	46.56; 40.54; 150.00; 119.30; 91.71; 68.88; 48.57; 46.44; 49.21	
Regional transit time in the GIT (h)	0.25; 0.26; 0.93; 0.74; 0.58; 0.42; 0.29; 4.13; 12.38	

**Table 4:** Comparison between GastroPlus^®^ simulated and in-vivo observed furosemide pharmacokinetic parameters following p.o. drug administration.

40 mg p.o. Dose					
Parameter	In-Vivo I ^a^	In-Vivo II ^b^	Predicted	PE(%) I	PE(%) II
C_max_ (µg/mL)	0.61	0.75	0.71	−17.14	5.54
t_max_ (h)	1.5	1.12	1.36	9.33	−22.22
AUC_0→∞_ (µg∙h/mL)	2.13	2.44	3.66	−71.25	−50.06
AUC_0→24 h_ (µg∙h/mL)	2.11	2.33	2.52	−19.25	−8.15
F (%)	NA	NA	52.2	NA	NA

^a^ Refers to the mean plasma profile from [43] (40 mg IR tablet); ^b^ refers to the mean plasma profile from [37] (40 mg IR tablet); NA, not available/not applicable.

**Table 5:** GastroPlus^®^ predicted pharmacokinetic parameters for different furosemide virtual dissolution profiles from 40 mg p.o. dosage forms.

Dissolution	C_max_ (µg/mL)	t_max_ (h)	AUC_0→__∞_ (µg∙h/mL)	F (%)
85% in 15 min	0.71	1.36	3.65	51.91
85% in 1 h	0.64	1.76	3.71	46.35
85% in 6 h	0.15	2.80	0.80	16.64
85% in 8 h	0.11	2.80	0.61	12.73
85% in 12 h	0.08	2.80	0.41	8.65
85% in 24 h	0.04	2.80	0.21	4.36
